# Study of the Physical, Chemical, and Structural Properties of Low- and High-Methoxyl Pectin-Based Film Matrices Including Sunflower Waxes

**DOI:** 10.3390/membranes13100846

**Published:** 2023-10-22

**Authors:** Mayra C. Chalapud, Ma. de la Paz Salgado-Cruz, Erica R. Baümler, Amalia A. Carelli, Eduardo Morales-Sánchez, Georgina Calderón-Domínguez, Alitzel B. García-Hernández

**Affiliations:** 1 Departamento de Ingeniería Química, Universidad Nacional del Sur (UNS), Bahía Blanca 8000, Argentina; mchalapud@plapiqui.edu.ar (M.C.C.); ebaumler@plapiqui.edu.ar (E.R.B.); acarelli@plapiqui.edu.ar (A.A.C.); 2 Planta Piloto de Ingeniería Química–PLAPIQUI (UNS-CONICET), Bahía Blanca 8000, Argentina; 3 Departamento de Ingeniería Bioquímica, Escuela Nacional de Ciencias Biológicas, Instituto Politécnico Nacional, Av. Wilfrido Massieu 399, Nueva Industrial Vallejo, Gustavo A. Madero, Ciudad de México 07738, Mexico; gcalderon@ipn.mx; 4 Centro de Investigación en Ciencia Aplicada y Tecnología Avanzada-Unidad Querétaro, Cerro Blanco No. 141, Col. Colinas del Cimatario, Santiago de Querétaro 76090, Mexico; emoraless@ipn.mx; 5 Consejo Nacional de Humanidades Ciencias y Tecnologías-Centro de Investigación en Química Aplicada-Unidad Monterrey, Parque de Investigación e Innovación Tecnológica, Autopista al Aeropuerto KM 10, Av. Alianza Sur 303, Ciudad Apodaca 66647, Mexico; ali_ialee@outlook.com

**Keywords:** polymer casting, biopolymers, gas barrier properties, mechanical properties

## Abstract

The development of bio-based materials remains one of the most important alternatives to plastic materials. Although research in this field is growing, reporting various materials and methodologies, it is still necessary to increase exploration. The aim of this work was to expand and complement previous research on the preparation and characterization of high- and low-methoxyl pectin films obtained by casting, with the addition of commercial and recovered sunflower waxes. The results showed that the addition of sunflower waxes to the pectin matrix generated some discontinuity in the aggregate, increasing the thickness and roughness of the film. However, due to their hydrophobic nature, the waxes contributed to lower vapor transmission rate values of the films. On the other hand, the low-methoxyl pectin films had a more crystalline structure, which could help to diminish water vapor permeability values, mechanical resistance and rigidity, and improve their elongation. Regarding chemical characteristics, most of the raw materials’ chemical groups were found in the resulting films, and the presence of C-H bending due to pectin gelation was observed. Finally, the compatibility and contribution of pectin and sunflower waxes to the production of the films were demonstrated, as well as the possibility of using materials from industrial waste in food packaging applications.

## 1. Introduction

Plastic materials, which are extensively utilized in food packaging, are mainly non-renewable and non-degradable products derived from petroleum compounds. For these reasons, the development of food packaging materials based on the use of components and polymers from natural sources has recently captured the attention of many researchers due to its ability to produce renewable and biodegradable coating materials [1]. For this purpose, natural raw materials such as polysaccharides, proteins and lipids and their combinations have been tested in packaging applications [2,3]. 

An example of these materials is pectin, which is one the most abundant structural polysaccharides in plant cells. As a raw material, pectin shows good functional properties; it has been suitable for use in the food, cosmetics and pharmaceutical industries as well as being one of the main substances tested for food packaging [4,5]. The properties of pectin are highly dependent on the degree of methyl esterification (DM, percentage of esterified carboxyl groups to total carboxyl groups in pectin) [6]. For example, high-methoxyl pectin (HMP, DM ≥ 50%) forms gels under acidic conditions, while low-methoxyl pectin (LMP, DM < 50%) needs positive ions (Ca^2+^). Pectin has found multiple applications in the food and pharmaceutical industries as a functional additive (drug delivery, cancer therapy, thickener, emulsifier and stabilizer, among others) as well as in edible films and coatings [7,8]. However, despite its use promoting the development of edible films as an environmentally friendly alternative, this polysaccharide continues to present mechanical and barrier deficiencies; therefore, it is frequently combined with hydrophobic substances such as waxes to improve barrier properties [7,8]. Moreover, valuable information has been collected about sunflower waxes recovered from the process of oil winterization, evaluating their incorporation as a reinforcement in food films [9,10,11]; therefore, there is growing research in the field of the continuous improvement of film-forming materials.

In this context, to identify current trends and collect specific data from research that analyzes the production of pectin-based films and the incorporation of sunflower waxes, a structured bibliometric analysis was carried out using specific keywords (low-methoxyl and high-methoxyl pectin, sunflower, waxes, films). In this search, few studies addressing the proposed research question were found, of which the first most-cited paper shows the physical and chemical characteristics and stability of film-forming solutions from low-methoxyl pectin and recovered sunflower waxes [12]. A second article, published in 2020, focuses on the formulation and characterization of pectin emulsions [13].

Meanwhile, the last two papers, by Chalapud et al. [14,15], were aimed at film elaboration. They differ not only in the filming techniques used (casting and electrospraying, respectively), but also in the fact that the first report studied low-methoxyl pectin films with varying pectin/wax proportions, and the second study also analyzed high-methoxyl pectin films using a single ratio of raw materials. However, it has been reported that the degree of methyl esterification (DM) had an impact on changes in the barrier and mechanical properties of pectin films due to the differences in gelling behavior.

Given this background, it is still not clear if the mechanical characteristics and barrier properties of films occur due to the interaction between the chemical structures of the different constituents, namely of the functional groups of waxes (esters having between 36 and 48 carbon atoms, fatty acids and fatty alcohols) and pectin (carboxyl groups esterified), or due to the method of production which modifies the film structure [16]. At this point, two main processes have been identified in the elaboration of edible films: (1) the “wet process, or casting method” and (2) the “dry process, or compression molding and extrusion”, the former being the cheapest and most environmentally friendly, and being widely applied for coating foods such as fruits [17,18] and confectionery products [19] or delivering probiotics [20]. For this reason, it is important to exploit this methodology and evaluate its advantages. 

In this sense, to expand the analysis of this area, this work evaluated the effect of the type of pectin and sunflower waxes on the composition, barrier and mechanical properties of films developed by casting using high-methoxyl and low-methoxyl pectin and commercial sunflower waxes.

## 2. Materials and Methods

### 2.1. Materials

Recovered sunflower waxes (RWs), a by-product derived from refining sunflower oil, were previously fractionated, purified and characterized as described by Chalapud et al. [9]. RWs were between 40 and 60 carbon atoms, with fatty alcohols between 18 and 34 and fatty acids between 14 and 34 carbon atoms, respectively [9]. Commercial sunflower waxes (CWs) were provided by Koster Keunen, USA. [21]. Low-methoxyl pectin (LMP) was purchased from CP Kelco, Argentina (GENU PECTIN type LM 104 AS, esterification degree 27%, and 20% of original carboxyl groups replaced by amide groups). High-methoxyl pectin (HMP) from citrus peel was obtained from Sigma-Aldrich (P9135, Mexico, 74% galacturonic acid content). NaBr and CaCl_2_ were acquired from J.T. Backer Inc., Phillipsburg, NJ, USA (98–99% purity, analytical grade reagent). Glycerol (G5516) (98–99% purity, analytical grade reagent) and Tween 20 (P1379) were used as plasticizer and surfactant, respectively (Sigma-Aldrich, Toluca, Mexico). 

### 2.2. Film-Forming Solutions and Emulsions

Pectin solutions from HMP or LMP without waxes were prepared with distilled water (2 g 100 g^−1^) until dissolved. Then, glycerol (50 g per 100 g of pectin) and Tween 20 (1:10, Tween 20/pectin) were added. The procedure proposed by Chalapud et al. [15] was followed to prepare the emulsions. Briefly, the pectin solution was heated at 85 °C in a water bath, and then, sunflower waxes (CWs or RWs) were added at 0.2 g per g of pectin. The emulsions were homogenized at 13,000 rpm for 10 min using an Ultra Turrax (T25 Basic IKA Labortechnik, Staufen, Germany) while keeping the temperature at 85 °C. Finally, CaCl_2_ was added at 1 g 100 g^−1^ pectin to low-methoxyl pectin emulsions to induce gel network formation in the presence of calcium ions.

### 2.3. Film Elaboration by Casting Method

Films were produced by the casting method, as suggested by Chalapud et al. [14], with some modifications. Approximately 6 g of the pectin solutions and emulsions obtained in Section 2.2 was poured into glass Petri dishes 10.5 cm in diameter and then dried in an air oven at 50 °C for 15 h. The resulting films were removed from the dishes and finally stored at room temperature in a saturated NaBr solution at 57% relative humidity for the next test. 

HMP and LMP films, elaborated from HMP and LMP solutions without waxes, respectively, were used as controls. CW-HMP and RW-HMP or CW-LMP and RW-LMP indicate films elaborated with CWs and RWs and with HMP and LMP, respectively. 

### 2.4. Film Characterization

#### 2.4.1. Film Thickness

The thickness of the films was measured using a digital micrometer (ID-C112EXB, Mitutoyo Corp, Japan). In summary, five measurements were taken at random from the central area to the periphery of the film. The average ± standard deviation was reported and was used to determine mechanical properties and permeability values.

#### 2.4.2. Film Microstructure 

The microstructure of the films was evaluated by scanning electron microscopy (SEM). Samples were prepared by cutting and were then gold-covered on a Denton Vacuum Desk II (United States). The micrographs were taken at 2000× under 15 kV of accelerating voltage, using a JEOL JSM 6460 LV (JEOL Ltd., Akishima, Japan). Surface and cross-sectional images were obtained.

#### 2.4.3. Atomic Force Microscopy (AFM)

The surface characteristics of the films was evaluated from surface topographic images obtained in three dimensions with real-time resolution in an atomic force microscope (diMultimode V, Veeco, Santa Barbara, CA, USA). The analysis was performed on a resonance frequency of 286–362 kHz and a constant force of 20–80 Nm^−1^. Then, each sample was cut into sections of 0.5 cm × 0.5 cm and two areas of 10 μm × 10 μm were scanned at 1 Hz with a resolution of 256 × 256 pixels. Quantitative data such as roughness (*R_q_*, Equation (1)) and the arithmetic means of the absolute values of the deviation heights (*R_a_*, Equation (2)) were obtained from topographic images using NanoScope Analysis 1.5 software (Veeco, Santa Bárbara, CA, USA) with a seven-degree flattening process. Determinations were carried out in triplicate.

(1) $$R_{q}=\sqrt{\frac{\sum {\left(Z_{i}\right)}^{2}}{N}}$$

(2) $$R_{a}=\frac{1}{N}\sum_{i=1}^{N}\left|Z_{i}\right|$$

where *Z_i_* is the height deviation from the mean of the heights, and *N* is the number of points in the image. 

#### 2.4.4. X-ray Diffraction

X-ray diffraction measurements were carried out in a powder diffractometer (Miniflex 600, Rigaku) working at 45 kV and 40 mA, using a Cu radiation source, filtered to Kα wavelength of 1.54 Å, from 3° to 35° in 2*θ*. The degree of crystallinity (*DC*%) was calculated using Equation (3). The crystalline and amorphous peak areas were separated and integrated using PeakFit software version 4.12 (SYSTAT, San Jose, CA, USA).

(3) $$DC\left(\%\right)=\frac{A_{c}}{A_{c}+A_{a}}+100$$

where *A_c_* is the integrated intensity of the crystalline phase and (*A_c_* + *A_a_*) is the total area of the diffractogram.

#### 2.4.5. Raman Spectroscopy

Raman spectra were obtained by LabRam HR800 (Horiba Jobin Yvon, Miyano-higashi, Kyoto, Japan), equipped with an optical microscope (Olympus, BX 41, Tokyo, Japan) with a 100× objective and a Peltier-cooled charged detector (temperature detector), with three excitation lines (532 nm, 633 nm and 785 nm) and an output power of 43.4 mW, 86.3 mW and 56.7 mW, respectively. Instrument calibration was conducted using a 520.5 cm^−1^ silicon line. Each sample was analyzed in triplicate, using 400 lines per millimeter and an emission laser of 785 nm, from 100 cm^−1^ to 3200 cm^−1^, at room temperature, an exposure time of 4–60 s and 10–20 scans. LabSpec V.6 (Horiba Jobin Yvon, Longjumeau, France) and OriginLab V.9.8 (OriginLab Corporation) software were used for data processing.

#### 2.4.6. Water Vapor Permeability (*WVP*) and Water Vapor Transmission Rate (*WVTR*)

Gas barrier properties (*WVP* and *WVTR*) were determined according to ASTM E96/E96M-05 [22], with some modifications. For this assessment, circular samples of films 3.3 cm in diameter were positioned on top of water-containing permeation cells (100% RH). The cells were placed into a controlled environment at 30 °C and zero % RH. For 24 h, cell weight was recorded every minute on an analytical balance (TP-214, Denver Instruments, Denver, CO, USA). Finally, *WVP* and *WVTR* were calculated according to Equations (4) and (5):

(4) $$WVTR=\frac{W_{L}}{t\times A}$$

(5) $$WVP=\frac{WVTR\times L}{∆P}$$

In Equation (4), $W_{L}$ is water loss in kg, *t* is time in s and *A* is area, considered as exposed film area (m^2^). Here, $\frac{W_{L}}{t}$ was obtained from the slope, calculated by linear regression. *WVP* was determined according to the Fick and Henry laws for gas diffusion through films (Equation (5)), where *L* is membrane thickness (m) and Δ*P* is partial water vapor pressure difference (kPa) on both sides of the film.

#### 2.4.7. Mechanical Properties

Tensile strength (TS) and elongation at break (E%) were evaluated according to ASTM D882-09 [23] using a TexturePro CT V1.6 Build (Brookfield Engineering Labs, Middleborough, MA, USA) and applying the methodology described by Valdespino-León et al. [24]. Films of 2.5 cm × 12.5 cm were placed in the double-handled set of the instrument. The test was carried out in triplicate with independent samples, with a speed of 0.3 mm s^−1^. TS and %E were obtained and Young’s modulus (ε) was calculated as the slope of the straight line (stress vs. strain) in the elastic zone behavior of the film, which was visually assessed. 

#### 2.4.8. Statistical Analysis

The results were presented as mean values with standard deviation. Significant differences in each test were analyzed using two-way ANOVA and Fisher test (α = 0.05). The factors considered were the type of pectin (HMP and LMP) and sunflower waxes (CWs and RWs). All statistics were performed using InfoStat software, version 2009 [25].

## 3. Results and Discussion

### 3.1. Film Characterization

Table 1 shows the average thickness of the pectin films. Control films (HMP, LMP) showed the lowest thickness values, which were significantly different compared with films with added sunflower waxes (*p* < 0.05). As expected, the addition of mass, represented by the inclusion of sunflower waxes, resulted in significantly higher thickness values (*p* < 0.05). It is worth noting that de-esterified carboxyl group content in low-methoxyl pectin is essential for the formation of a highly ordered, mechanically stable “egg-box” structure, according to Çavdaroğlu et al. [26], which can generate a thinner structure than high-methoxyl pectin (Table 1). On the other hand, the chains of high-methoxyl pectin could then be linked through hydrophobic interactions with the methoxyl groups or through hydrogen bonds, including those of non-ionized acidic groups, which is why when adding waxes to this type of pectin, the thickness increases, compared to that of low-methoxyl pectin. It is important to mention that the interactions between the different types of pectin and the waxes were crucial, in that they jointly generated a change in thickness as well as rigidity and crystallinity structure. No significant differences were found between the films elaborated with each type of pectin (*p* > 0.05).

### 3.2. SEM Microscopy

**Figure 1:**
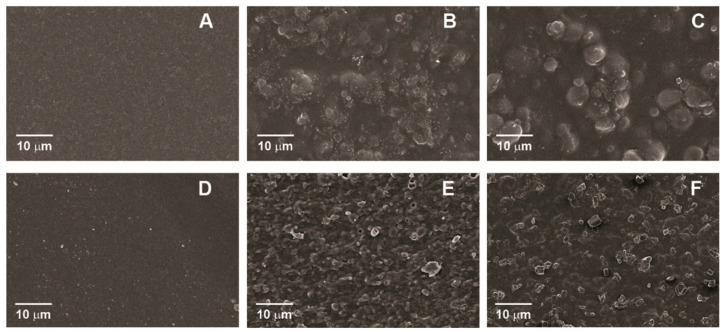
Top surface of pectin films: (**A**) high-methoxyl pectin (HMP), (**B**) commercial wax–HMP, (**C**) recovered wax–HMP, (**D**) low-methoxyl pectin (LMP), (**E**) commercial wax–LMP and (**F**) recovered wax–LMP.

**Figure 2:**
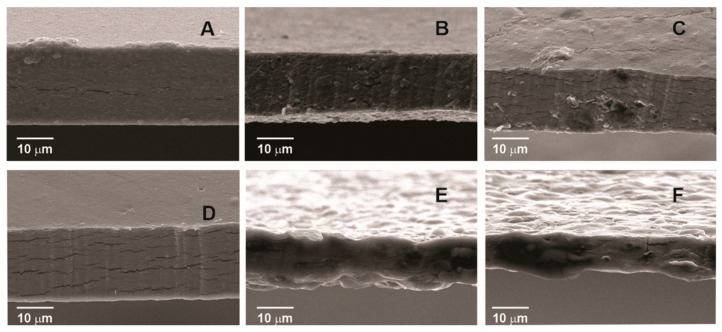
Cross-section of pectin films: (**A**) high-methoxyl pectin (HMP), (**B**) commercial wax–HMP, (**C**) recovered wax–HMP, (**D**) low-methoxyl pectin (LMP), (**E**) commercial wax–LMP and (**F**) recovered wax–LMP.

SEM micrographs of control pectin films (HMP and LMP) and those with added sunflower waxes are presented in Figure 1 and Figure 2 (top surface and cross-sectional area, respectively). In Figure 1A,D, a uniform defect-free surface can be observed in the control films, while those containing sunflower waxes (Figure 1B,C,E,F) present discontinuities caused by the presence of CWs and RWs as small structures and their aggregates. Compared to HMP control films, some cracks are observed along the cross-section of the LPM control films (Figure 2D). These structural differences between LMP and HMP control films can be attributed to interactions between low-methoxyl pectin and Ca^2+^ ions during gel formation, resulting in a different internal arrangement. Moreover, aggregates of sunflower waxes are also visible in cross-sectional images (Figure 2B,C,E,F). Regarding pectin type, films prepared with LMP display a less uniform surface, with some degree of network and the presence of some crystals. These crystalline structures may correspond to salts formed with the chlorine present in CaCl_2_ [27], which were slowly growing after the addition of calcium chloride to the film-forming solution and the subsequent drying process [28]. This result differs from that reported in previous publications [14,15], where the time in which the crystals could have formed was shorter and the methodology employed was different; therefore, the presence of crystals was not evidenced. Different morphologies, evidenced in SEM cross-sectional images (Figure 2), resulted from structural arrangements of the films, due mainly to the different gelation mechanisms of two types of pectin. A Ca^2+^-pectin gel network is produced in LPM films by “junction zones,” whose development mechanism is mostly based on the “egg-box” model. The addition of CWs and RWs to the network films may have resulted in junction zones that were weaker, fewer in number, shorter and less stable [29]. Comparing this structure to HMP films, it can be observed that it is less ordered and less firm, which may affect film strength.

### 3.3. Atomic Force Microscopy (AFM)

**Figure 3:**
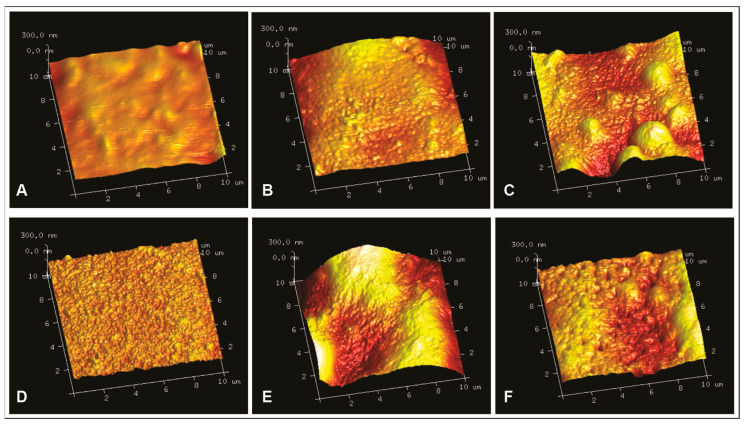
AFM 3D images of pectin films: (**A**) high-methoxyl pectin (HMP), (**B**) commercial wax–HMP, (**C**) recovered wax–HMP, (**D**) low-methoxyl pectin (LMP), (**E**) commercial wax–LMP and (**F**) recovered wax–LMP.

**Figure 4:**
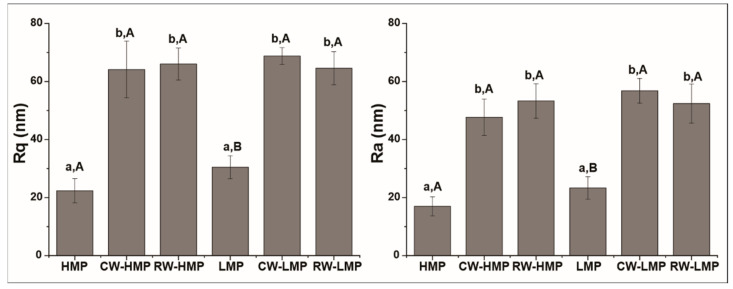
Roughness mean values (*R_q_* and *R_a_*) of films made of high-methoxyl pectin (HMP), commercial waxes (CW) –HMP, recovered waxes (RW) –HMP, low-methoxyl pectin (LMP), CW–LMP and RW–LMP. Mean values ± standard deviation. n = 3. Different lowercase letters (effect of wax addition on films based on the same type of pectin) and different uppercase letters (effect of type of pectin) are significantly different (*p* < 0.05) in the Fisher test.

The light and dark regions of the AFM images represent the peaks and valleys of the pectin films, respectively. As expected, and observed by SEM, surface irregularities were reduced and the topography was smoother in the control films (HMP and LMP, Figure 3A,D) than in those containing sunflower waxes, which is reflected in significantly lower values of *R_q_* and *R_a_* (*p* < 0.05) (Figure 4). AFM images revealed differences in the surfaces of both control films (Figure 3A,D). The lighter areas in LMP films could indicate the junction zones formed due to the binding between Ca^2+^ and units of non-esterified galacturonic acids of low-methoxyl pectin, which is described in the “egg-box” model [29,30], and, therefore, a significantly rougher surface was obtained (*p* < 0.05). These results would suggest that the hydrophobic interactions and hydrogen bonds involved in HMP film formation were more effective in obtaining smoother films, compared to Ca^2+^ cross-linking for LMP films. While HMP roughness values were higher than those reported by Priyadarshi et al. [31] and Gaona Sánchez et al. [32], those obtained for LMP were lower than those presented by Sartori et al. [33]. Likewise, when CWs and RWs were incorporated into the films, AFM images showed brighter areas (Figure 3B,C,E,F), and, therefore, *R_q_* and *R_a_* values increased significantly (Figure 4) (*p* < 0.05). However, there are no significant differences between wax-containing films based on the same type of pectin (CW-HMP and RW-HMP; CW-LMP and RW-LMP) (*p* > 0.05). This significant increase in roughness in HMP films with added sunflower waxes can be attributed to the lower degree of packing [31]. On the other hand, compared to previous studies, the roughness values of all pectin films were higher than those obtained by electrospraying [15], indicating that the surface roughness of films is influenced by the method in which they were processed [32].

### 3.4. X-ray Diffraction

**Figure 5:**
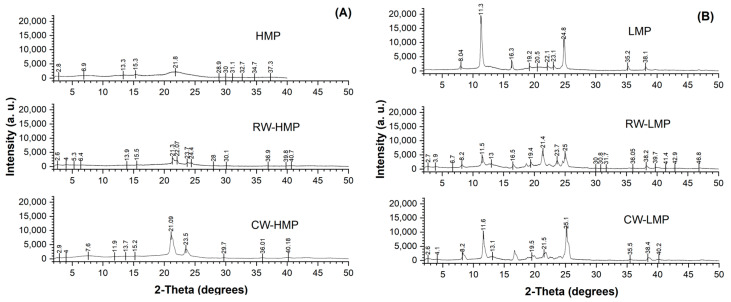
X-ray diffraction patterns of (**A**) only high-methoxyl pectin (HMP), HMP with recovered sunflower waxes (RWs) and HMP with commercial sunflower waxes (CWs) and (**B**) only low-methoxyl pectin (LMP), RW-LMP and CW-LMP.

The diffractogram corresponding to HMP films (Figure 5A) presents an intense peak at 21.8° in 2*θ* (d-spacing 4.05 Å), which, according to Valdespino-León et al. [24], is associated with an amorphous portion formed by esterified carboxyl groups. This peak disappears in LMP films (Figure 5B), increasing their crystallinity, as shown in Table 2. Regarding films prepared with high-methoxyl pectin with RWs and CWs (Figure 5A), they present diffraction patterns with characteristic peaks at 21.3°, 22.07°, 23.7°, 24.4°, 21.09° and 23.5° in 2*θ* with interplanar distances of 4.15, 4.02, 3.74, 3.64, 4.21 and 3.78 Å, considered as “short spacing peaks”, according by Bharti et al. [34]. It should be noted that in films with added commercial and recovered waxes, these short-spaced peaks are narrower and more defined; this behavior could be related to more organized structures due to the packing degree of hydrocarbon chains in three-dimensional pectin networks. However, the global crystallinity of films decreases, despite the fact that the sunflower wax peaks are still maintained in the mixture as two ordered peaks in a crystalline position, according to what was reported by Chalapud et al. [15]. These results are important because the configuration of the wax crystals embedded in the polymeric matrix determines the mechanical and structural characteristics of the films. 

Regarding films elaborated with low-methoxyl pectin (Figure 5B), the control film (LMP) presents three well-defined peaks at 11.3, 16.3 and 24.8° (d spacing 7.82, 5.43 and 3.59 Å, respectively) in 2*θ* (Figure 5B). As observed in Table 2, all LMP films presented a significantly higher *DC* % compared to that of HMP films (*p* < 0.05). These results agree with a previous study [15], where low-methoxyl pectin raw material had a more crystalline structure. Despite the peaks of sunflower wax being in crystalline positions [15], the *DC* % of LMP films decreased significantly with the incorporation of RWs or CWs (*p* < 0.05). However, the peaks at 19.5, 21.5 and 25.1 in 2*θ* in CW-LMP films become more evident. The decrease in the degree of crystallinity of films when CWs and RWs are incorporated may suggest that, due to their addition into the mixture as melted components, a new state of wax recrystallization was produced [35] in conjunction with a conformational change to their amorphous structure, which impacted the crystallinity of the films. In this context, the new conformational arrangement of film components could be related to the variation in the water vapor transmission rate (*WVTR*) and water vapor permeability (*WVP*) values. 

### 3.5. Raman Spectroscopy

**Figure 6:**
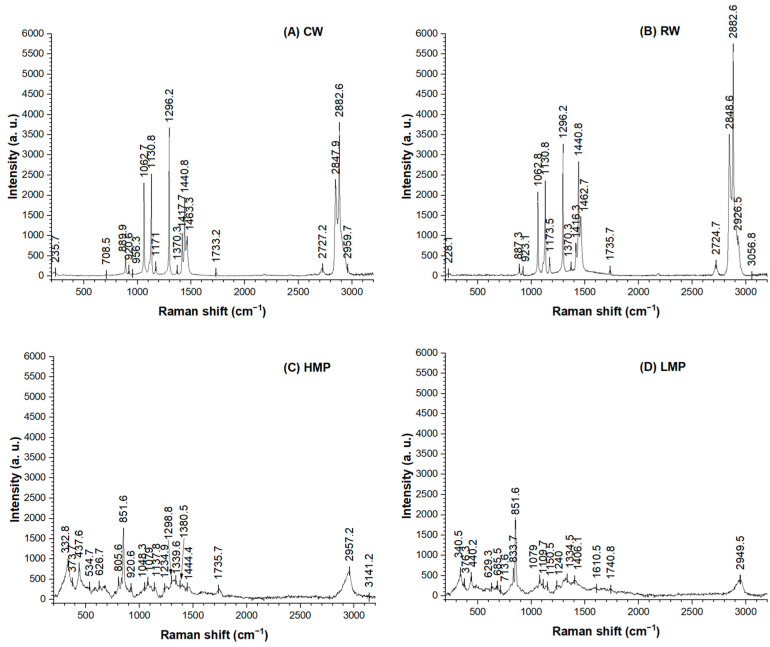
Confocal Raman spectra of raw materials: (**A**) commercial sunflower waxes (CWs), (**B**) recovered sunflower waxes (RWs), (**C**) high-methoxyl pectin (HMP) and (**D**) low-methoxyl pectin (LMP).

**Figure 7:**
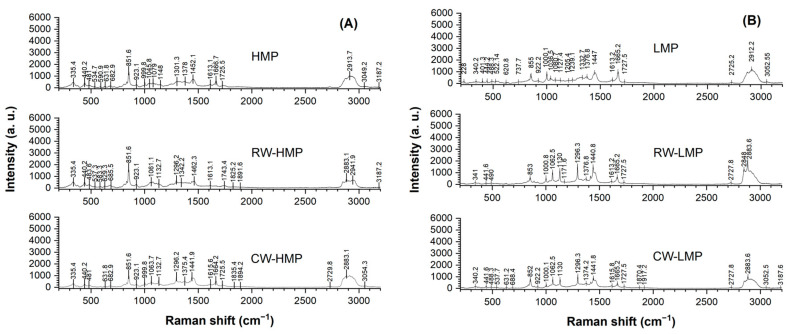
Confocal Raman spectra of (**A**) high-methoxyl (HMP) and (**B**) low-methoxyl pectin (LMP) films. Control films with only HMP and LMP, with recovered sunflower waxes (RWs) and with commercial sunflower waxes (CWs).

Raman microscopy was used to assess the structural homogeneity between the waxes (CWs and RWs) and pectin, as well as to examine potential changes in the component distribution of films produced by the casting method. Raman spectra of the raw materials (CWs, RWs, HMP and LMP) are presented in Figure 6. On the other hand, Figure 7 shows the characteristic vibrational modes of the chemical groups of high and low-methoxyl pectin films, respectively. All pectin films presented a C-H stretch vibration signal at 2900 cm^−1^ for pectin [36]. This C-H band of pyranosed ring carbons shifted to 2957 and 2949 cm^−1^ for HMP and LMP raw materials, respectively, and to 2915 and 2913 cm^−1^ for HMP and LMP films, respectively. In the film spectra (Figure 7), a weak band is distinguished at around 1376 cm^−1^ and a peak is missing in the raw pectin spectrum (Figure 6C,D), suggesting the generation of a C-H bending in the films as a result of the gelation of both types of pectin [37]. Additionally, in relation to polygalacturonic acid, the band at 1080 cm^−1^, which appears in all film spectra with a similar intensity, could be assigned to the C-O and O-H deformation vibration [37]. Furthermore, all spectra show a notable band around 850 cm^−1^, associated with the presence of galacturonic acid and glycosidic bonds (C-O-C asymmetric stretch) in pectin [37,38]. Also, vibrational bands between 837 and 875 cm^−1^ are related to the arrangement of a pectin ring with CH bonds, indicating that pectin is present around other structures [39]. In addition, the slight shift observed at 441 cm^−1^ is assigned to the C-O-C torsion deformation of methyl polygalacturonate [40] found in pectin raw material (Figure 6C,D) and its films (Figure 7). Furthermore, the wax raw materials (Figure 6A,B) show similar spectral results, having common bands at 1062, 1131 and 1295 cm^−1^, which correspond to ν(CO), ν(CC) and ν(CH_2_) twisting vibrational modes, respectively, and are essential for the identification of unsaturated waxes such as those used in this work [9,41]. On the other hand, some bands at 2881 cm^−1^, 2848 cm^−1^ and 1418–1463 cm^−1^ found in CWs and RWs (Figure 6A,B) are maintained in pectin films, corresponding to asymmetric vibrations of the ν(CH_2_), ν(CH_3_-CH) and δ(CH_2_) bands, respectively [41]. These bands were also observed in all films with decreased intensity due to the interaction with the raw materials, and, as expected, they were absent in control pectin films (HMP and LMP in Figure 7).

Regarding RW-HMP, a band at 1744 cm^−1^ is characteristic of the carboxyl ester vibration (C=O) that determines the pectin methyl esterification degree [38,42]. This band is absent in the HMP control film, CW-HMP and films elaborated with low-methoxyl pectin, which could present a displacement at 1727 cm^−1^ due to the carbonyl groups derived from fatty alcohols and fatty acids contained in sunflower waxes. These modifications could also indicate a lower degree of esterification and a lower cross-linking capacity, affecting the film’s mechanical properties.

### 3.6. Water Vapor Transmission Properties of Films

The water vapor transmission rate (*WVTR*) and water vapor permeability (*WVP*) values of pectin films are shown in Table 3. The *WVTR* showed a significant reduction with the incorporation of sunflower wax in high- and low-methoxyl pectin films, a behavior attributed to the hydrophobic character of the waxes. A significant reduction (*p* < 0.05) was obtained by adding RWs to LMP films. These results are in agreement with previous works [14,15], where a comparable WVRT reduction was reported when using sunflower waxes on pectin films. Other waxes such as beeswax, candelilla, carnauba waxes and *Terminalia catappa* Linn. leaf wax have shown similar behavior when added to films [43,44,45]. In contrast, *WVP* values behaved differently, as the incorporation of sunflower waxes into pectin films increased *WVP* values compared to control films. This fact can be explained by the positive correlation between *WVP* and the increase in film thickness (Equation (4)), but also by the discontinuities caused by the development of sunflower wax aggregates, which produced areas in the films with higher roughness (as observed by SEM and AFM images). Also, discontinuities can cause a lack of cohesion between the film components (pectin and waxes) in these areas, giving rise to microcracks around small drops of wax that increase the flow of water vapor, as reported by de Oliveira Filho et al. [43], who stated that the *WVP* values of films produced by microemulsions of carnauba waxes and arrowroot starch were affected by the discontinuities promoted by the aggregated wax.

The structure of the film and its barrier properties are affected by differences in the gelation mechanism of each type of pectin, as was already mentioned in a previous work [15]. The *WVTR* and *WVP* values of LMP films were lower than those obtained for HMP, and significant differences were found when incorporating CWs and RWs (*p* < 0.05). It can be seen that the “egg-box” structure developed with LMP [46] combined with sunflower waxes resulted in a more stable network for water vapor flow, while adding an “apparent tortuosity” to water molecule migration, than the film structure formed by HMP. In addition, the structure with the highest degree of crystallinity found in the films elaborated with LMP (Table 2) may contribute to a decrease in the flow of water vapor.

### 3.7. Mechanical Properties of Pectin Films

The mechanical properties of the films are shown in Table 4. All films produced with HMP were more resistant than those prepared with LMP, with significantly higher TS values (*p* < 0.05). These results are related to those observed in SEM images, where the internal structural arrangement of LMP films caused the network generated by cross-linking with Ca^2+^ ions to be less resistant [47] despite achieving the highest degree of crystallinity. This fact was also reflected in higher **ε** values in HMP films, where the structure formed by high-methoxyl pectin and sunflower waxes was stiffer, a characteristic that significantly increased with wax addition (*p* < 0.05). However, for each type of pectin, the addition of sunflower waxes did not have a significant impact on TS (*p* > 0.05). LMP films with CWs and RWs presented higher %E values, with significant differences being found only between films produced with RWs (*p* < 0.05). These results correlate with the high crystallinity of the LMP films (Table 2), where a high chain mobility allowed us to obtain films with high %E and low TS. This inverse relationship between TS and %E associated with the degree of crystallinity has been reported in chitosan/starch and gelatin/cornstarch films [2,3]. The protection and handling resistance of packaging materials are determined by their tensile strength, whereas their capacity to resist shape changes without cracking is determined by their elongation. Without ignoring the good mechanical properties attained with films produced with low-methoxyl pectin, in this context, films elaborated with high-methoxyl pectin and the addition of sunflower waxes (CWs and RWs) could satisfy both features.

## 4. Conclusions

The casting method is the most suitable for obtaining films elaborated with high- and low-methoxyl pectin and sunflower waxes (commercial and recovered) with good mechanical and structural qualities. A structural analysis of the films allowed us to visualize the sunflower wax aggregates distributed over the entire cross-sectional view of the films and to identify the morphological differences between LMP and HMP films caused by their different internal arrangements. These structural differences were related to the mechanical properties of the films. As a result, HMP films with added sunflower waxes were more suitable for a mechanical food-protective function. Chemical and structural identification by the Raman method showed that the main vibrational signals present in the raw materials (pectin and sunflower waxes) were maintained in the films, with varying intensities and shifting depending on the structure and interactions of the film components. Although the high crystallinity of LMP films contributed to the reduction in water vapor permeability, the incorporation of sunflower waxes into the pectin matrix reduced the water vapor transmission rate, but due to the discontinuities, water vapor permeability values increased.

## Figures and Tables

**Table 1:** Thickness of high-methoxyl and low-methoxyl pectin films.

Film	Thickness (µm)	Film	Thickness (µm)
HMP	^A^ 22 ± 3 ^a^	LMP	^A^ 21 ± 4 ^a^
CW-HMP	^A^ 35 ± 2 ^b^	CW-LMP	^A^ 32 ± 4 ^b^
RW-HMP	^A^ 33 ± 4 ^b^	RW-LMP	^A^ 31 ± 8 ^b^

Mean values ± standard deviation. n = 5. Values in the same column followed by different lowercase letters (effect of adding sunflower waxes) and values in the same row preceded by different uppercase letters (effect of type of pectin) are significantly different (*p* < 0.05) in the Fisher test. HMP: high-methoxyl pectin, LMP: low-methoxyl pectin, CW: commercial sunflower wax and RW: recovered sunflower wax.

**Table 2:** Degree of crystallinity (*DC*%) of high-methoxyl and low-methoxyl pectin films.

Film	*DC* (%)	Film	*DC* (%)
HMP	^A^ 4.4 ± 1.2 ^a^	LMP	^B^ 50.4 ± 4.6 ^c^
CW-HMP	^A^ 3.6 ± 0.9 ^a^	CW-LMP	^B^ 26.9 ± 3.0 ^b^
RW-HMP	^A^ 3.5 ± 1.7 ^a^	RW-LMP	^B^ 14.3 ± 0.4 ^a^

Mean values ± standard deviation. n = 2. Values in the same column followed by different lowercase letters (effect of adding sunflower waxes) and values in the same row preceded by different uppercase letters (effect of type of pectin) are significantly different (*p* < 0.05) in the Fisher test. HMP: high-methoxyl pectin, LMP: low-methoxyl pectin, CW: commercial sunflower wax and RW: recovered sunflower wax.

**Table 3:** Water vapor transmission rate (*WVTR*) and water vapor permeability (*WVTR*) of high-methoxyl and low-methoxyl pectin films.

Film	WVTR (kg·m^−2^·s^−1^)	*WVP* × 10^−7^ (kg·Pa^−1^·m^−1^·s^−1^)	Film	*WVTR* (kg·m^−2^·s^−1^)	*WVP* × 10^−7^ (kg·Pa^−1^·m^−1^·s^−1^)
HMP	^A^ 25.40 ± 1.17 ^a^	^A^ 13.10 ± 0.55 ^a^	LMP	^A^ 24.60 ± 1.64 ^c^	^A^ 12.20 ± 0.16 ^a^
CW-HMP	^B^ 23.90 ± 0.97 ^a^	^B^ 19.20 ± 0.27 ^b^	CW-LMP	^A^ 20.70 ± 0.19 ^b^	^A^ 15.60 ± 0.27 ^b^
RW-HMP	^B^ 22.80 ± 2.31 ^a^	^B^ 17.80 ± 0.13 ^b^	RW-LMP	^A^ 18.40 ± 0.83 ^a^	^A^ 13.30 ± 0.55 ^ab^

Mean values ± standard deviation. n = 3. Values in the same column followed by different lowercase letters (effect of adding sunflower waxes) and values in the same row preceded by different uppercase letters (effect of type of pectin) are significantly different (*p* < 0.05) in the Fisher test. HMP: high-methoxyl pectin, LMP: low-methoxyl pectin, CW: commercial sunflower wax and RW: recovered sunflower wax.

**Table 4:** Mechanical properties of high-methoxyl and low-methoxyl pectin films.

Film	%E	TS (MPa)	ε (MPa)
HMP	6.32 ± 0.47 ^a, A^	7.80 ± 0.58 ^a, B^	142.97 ± 2.44 ^a, B^
LMP	5.36 ± 0.20 ^a, A^	6.06 ± 0.40 ^a, A^	116.51 ± 8.27 ^a, A^
CW-HMP	6.59 ± 1.37 ^a, A^	8.55 ± 0.39 ^a, B^	280.68 ± 11.29 ^b, B^
CW-LMP	8.91 ± 0.13 ^b, A^	6.17 ± 1.02 ^a, A^	122.68 ± 3.94 ^a, A^
RW-HMP	4.79 ± 0.71 ^a, A^	8.20 ± 1.23 ^a, B^	289.61 ± 8.26 ^b, B^
RW-LMP	6.97 ± 1.02 ^a, B^	6.18 ± 0.02 ^a, A^	121.19 ± 4.74 ^a, A^

Mean values ± standard deviation. n = 3. Values followed by different lowercase letters (effect of adding waxes to films with the same type of pectin) and values in the same column followed by different uppercase letters (effect of type of pectin) are significantly different (*p* < 0.05) in the Fisher test. HMP: high-methoxyl pectin, LMP: low-methoxyl pectin, CW: commercial sunflower wax and RW: recovered sunflower wax.

## Data Availability

Not applicable.

## References

[1] Mellinas C., Ramos M., Jiménez A., Garrigós M.C. (2020). Recent trends in the use of pectin from agro-waste residues as a natural-based biopolymer for food packaging applications. Materials.

[2] Scopel B.S., Ribeiro M.E., Dettmer A., Baldasso C. (2018). Cornstarch-gelatin films: Commercial gelatin versus chromed leather waste gelatin and evaluation of drying conditions. J. Polym. Environ..

[3] Sun K.-Q., Li F.-Y., Li J.-Y., Li J.-F., Zhang C.-W., Chen S., Sun X., Cui J.-F. (2019). Optimisation of compatibility for improving elongation at break of chitosan/starch films. RSC Adv..

[4] Chaiwarit T., Ruksiriwanich W., Jantanasakulwong K., Jantrawut P. (2018). Use of orange oil loaded pectin films as antibacterial material for food packaging. Polymers.

[5] Nastasi J.R., Kontogiorgos V., Daygon V.D., Fitzgerald M.A. (2022). Pectin-based films and coatings with plant extracts as natural preservatives: A systematic review. Trends Food Sci. Technol..

[6] Cui Y., Chen J., Zhang S. (2023). The effect of degree of esterification of pectin on the interaction between pectin and wheat gluten protein. Food Hydrocoll..

[7] Cortés-Camargo S., Román-Guerrero A., Alpizar-Reyes E., Pérez-Alonso C. (2023). New Sources of Pectin: Extraction, Processing, and Industrial Applications. Utilization of Pectin in the Food and Drug Industries.

[8] Zhang M., Bai B., Cheng H., Ye X., Chang J., Chen S., Chen J. (2023). A method for gel grade determination and application evaluation of two citrus pectins. Int. J. Biol. Macromol..

[9] Chalapud M.C., Baümler E.R., Carelli A.A. (2017). Characterization of waxes and residual oil recovered from sunflower oil winterization waste. Eur. J. Lipid Sci. Technol..

[10] Baümler E.R., Carelli A.A., Martini S. (2013). Physical properties of aqueous solutions of pectin containing sunflower wax. J. Am. Oil Chem. Soc..

[11] Baümler E.R., Carelli A.A., Martini S. (2014). Preparation and physical properties of calcium pectinate films modified with sunflower wax. Eur. J. Lipid Sci. Technol..

[12] Chalapud M.C., Baümler E.R., Carelli A.A. (2018). Emulsions of sunflower wax in pectin aqueous solutions: Physical characterization and stability. Food Res. Int..

[13] Akkaya S., Ozel B., Oztop M.H., Yanik D.K., Gogus F. (2020). Physical characterization of high methoxyl pectin and sunflower oil wax emulsions: A low-field 1H NMR relaxometry study. J. Food Sci..

[14] Chalapud M.C., Baümler E.R., Carelli A.A. (2020). Edible films based on aqueous emulsions of low-methoxyl pectin with recovered and purified sunflower waxes. J. Sci. Food Agric..

[15] Chalapud M.C., Baümler E.R., Carelli A.A., Salgado-Cruz M.d.l.P., Morales-Sánchez E., Rentería-Ortega M., Calderón-Domínguez G. (2022). Pectin Films with Recovered Sunflower Waxes Produced by Electrospraying. Membranes.

[16] Mendes J., Norcino L., Manrich A., Pinheiro A., Oliveira J., Mattoso L. (2020). Characterization of pectin films integrated with cocoa butter by continuous casting: Physical, thermal and barrier properties. J. Polym. Environ..

[17] Kumar S., Reddy A.R.L., Basumatary I.B., Nayak A., Dutta D., Konwar J., Purkayastha M.D., Mukherjee A. (2023). Recent progress in pectin extraction and their applications in developing films and coatings for sustainable food packaging: A review. Int. J. Biol. Macromol..

[18] Panahirad S., Naghshiband-Hassani R., Mahna N. (2020). Pectin-based edible coating preserves antioxidative capacity of plum fruit during shelf life. Food Sci. Technol. Int..

[19] Shulga O., Hrybkov S., Shulga S. (2022). Ecological packaging materials for bakery and confectionery products based on a new pectin modification. Ukr. Food J..

[20] Shahrampour D., Khomeiri M., Razavi S.M.A., Kashiri M. (2020). Development and characterization of alginate/pectin edible films containing Lactobacillus plantarum KMC 45. LWT.

[21] Koster Keunen Sunflower Wax. https://www.kosterkeunen.com/product/sunflower-wax/.

[22] (2005). Standard Test Methods for Water Vapor Transmission of Materials.

[23] (2009). Standard Test Methods for Tensile Properties of Thin Plastic Sheeting.

[24] Valdespino-León M., Calderón-Domínguez G., De La Paz Salgado-Cruz M., Rentería-Ortega M., Farrera-Rebollo R.R., Morales-Sánchez E., Gaona-Sánchez V.A., Terrazas-Valencia F. (2021). Biodegradable electrosprayed pectin films: An alternative to valorize coffee mucilage. Waste Biomass Valorization.

[25] Di Rienzo J. (2009). InfoStat Versión 2009.

[26] Çavdaroğlu E., Büyüktaş D., Farris S., Yemenicioğlu A.J.F.H. (2023). Novel edible films of pectins extracted from low-grade fruits and stalk wastes of sun-dried figs: Effects of pectin composition and molecular properties on film characteristics. Food Hydrocoll..

[27] Assifaoui A., Loupiac C., Chambin O., Cayot P. (2010). Structure of calcium and zinc pectinate films investigated by FTIR spectroscopy. Carbohydr. Res..

[28] Nesic A., Meseldzija S., Onjia A., Cabrera-Barjas G. (2022). Impact of Crosslinking on the Characteristics of Pectin Monolith Cryogels. Polymers.

[29] Ngouémazong D.E., Tengweh F.F., Fraeye I., Duvetter T., Cardinaels R., Van Loey A., Moldenaers P., Hendrickx M. (2012). Effect of de-methylesterification on network development and nature of Ca^2+^-pectin gels: Towards understanding structure–function relations of pectin. Food Hydrocoll..

[30] Han W., Meng Y., Hu C., Dong G., Qu Y., Deng H., Guo Y. (2017). Mathematical model of Ca^2+^ concentration, pH, pectin concentration and soluble solids (sucrose) on the gelation of low methoxyl pectin. Food Hydrocoll..

[31] Priyadarshi R., Kim S.-M., Rhim J.-W. (2021). Pectin/pullulan blend films for food packaging: Effect of blending ratio. Food Chem..

[32] Gaona Sánchez V.A., Calderón Domínguez G., Morales Sánchez E., Chanona Pérez J.J., Arzate Vázquez I., Terrés Rojas E. (2016). Pectin-based films produced by electrospraying. J. Appl. Polym. Sci..

[33] Sartori T., Feltre G., do Amaral Sobral P.J., da Cunha R.L., Menegalli F.C. (2018). Properties of films produced from blends of pectin and gluten. Food Packag. Shelf Life.

[34] Bharti D., Kim D., Cerqueira M.A., Mohanty B., Habibullah S., Banerjee I., Pal K. (2021). Effect of biodegradable hydrophilic and hydrophobic emulsifiers on the oleogels containing sunflower wax and sunflower oil. Gels.

[35] Cruces F., García M.G., Ochoa N.A. (2021). Reduction of Water Vapor Permeability in Food Multilayer Biopackaging by Epitaxial Crystallization of Beeswax. Food Bioprocess Technol..

[36] Szymańska-Chargot M., Chylińska M., Pieczywek P.M., Rösch P., Schmitt M., Popp J., Zdunek A. (2016). Raman imaging of changes in the polysaccharides distribution in the cell wall during apple fruit development and senescence. Planta.

[37] Chylińska M., Szymańska-Chargot M., Zdunek A. (2016). FT-IR and FT-Raman characterization of non-cellulosic polysaccharides fractions isolated from plant cell wall. Carbohydr. Polym..

[38] Engelsen S.B., Nørgaard L. (1996). Comparative vibrational spectroscopy for determination of quality parameters in amidated pectins as evaluated by chemometrics. Carbohydr. Polym..

[39] Zhuang Y., Sterr J., Kulozik U., Gebhardt R. (2015). Application of confocal Raman microscopy to investigate casein micro-particles in blend casein/pectin films. Int. J. Biol. Macromol..

[40] Synytsya A., Čopíková J., Matějka P., Machovič V. (2003). Fourier transform Raman and infrared spectroscopy of pectins. Carbohydr. Polym..

[41] Edwards H., Falk M. (1997). Fourier-transform Raman spectroscopic study of unsaturated and saturated waxes. Spectrochim. Acta A Mol. Biomol. Spectrosc..

[42] Nogales-Bueno J., Baca-Bocanegra B., Rooney A., Hernández-Hierro J.M., Byrne H.J., Heredia F.J. (2017). Study of phenolic extractability in grape seeds by means of ATR-FTIR and Raman spectroscopy. Food Chem..

[43] de Oliveira Filho J.G., Bezerra C.C.d.O.N., Albiero B.R., Oldoni F.C.A., Miranda M., Egea M.B., de Azeredo H.M.C., Ferreira M.D. (2020). New approach in the development of edible films: The use of carnauba wax micro-or nanoemulsions in arrowroot starch-based films. Food Packag. Shelf Life.

[44] Muscat D., Adhikari R., McKnight S., Guo Q., Adhikari B. (2013). The physicochemical characteristics and hydrophobicity of high amylose starch–glycerol films in the presence of three natural waxes. J. Food Eng..

[45] Mehraj S., Sistla Y.S., Garg M., Santra B., Grewal H.S., Kanjilal A. (2023). Improvement of Moisture Barrier and Tensile Properties of Pectin Films by Incorporating Terminalia catappa Linn. Leaf Wax and Xylitol. J. Polym. Environ..

[46] Cao L., Lu W., Mata A., Nishinari K., Fang Y. (2020). Egg-box model-based gelation of alginate and pectin: A review. Carbohydr. Polym..

[47] Wei H., Pascall M.A. (2023). Evaluation of structural and functional properties of citrus pectin film enriched with green tea extract. Polym. Eng. Sci..

